# Mortality and return to work in patients transported by emergency ambulance after involvement in a traffic accident

**DOI:** 10.1186/s12873-020-00382-3

**Published:** 2020-11-12

**Authors:** Kristian Bundgaard Ringgren, Elisabeth Helen Anna Mills, Erika Frischknecht Christensen, Rikke Nørmark Mortensen, Christian Torp-Pedersen, Kristian Hay Kragholm

**Affiliations:** 1 Department of Cardiology, Aalborg University Hospital, Aalborg, Denmark; 2 Center for Prehospital and Emergency Research, Department of Clinical Medicine Aalborg University and Clinic of Internal and Emergency Medicine Aalborg University Hospital, Aalborg, Denmark; 3 Unit of Epdimemiology and Biostatistics, Aalborg University Hospital, Aalborg, Denmark; 4 Department of Clinical Research, Nordsjællands Hospital, Hillerød, Denmark

**Keywords:** Road traffic injury (RTI), Road traffic accident (RTA), Return to work (RTW), Long-term outcome

## Abstract

**Introduction/background:**

Traffic accidents constitute a common reason for injury. Little is known about long-term outcomes for patients following a traffic accident. Therefore, in this present paper, we examine 1-day, 30-day and 1-year mortality, and return to work (RTW) during a 1-year period.

**Methods:**

Patients (between 18 and 65 years of age) who had an ambulance dispatched to them following a traffic accident and who were employed prior to the accident were identified from the Electronic Prehospital Emergency Patient (amPHI™) database in the North Denmark Region (catchment population ≈600,000) during 2006–2014. Outcomes of 1- and 30- and 365-day mortality and 1-year return to work (RTW), with mortality as competing risk. We stratified by intensive care unit (ICU) admission; and the anatomical region of injury (head/neck, thorax, abdomen, extremities and multiple injuries) is reported.

**Results:**

Of 6072 patients in our study population, 59 (1%) died within 1 day and 76 (1.3%) within 30 days; 88 (1.5%) were dead within a year.

Thirty-day mortality was 1.7% for the 290 patients admitted to the ICU, and 1.2% for the remaining 5782 patients.

Within the study population, RTW rate was 92.7% (*N* = 5984). RTW was 84.8% among 290 ICU-admitted patients versus 93.1% for the remaining 5782 patients.

RTW rate was 94.6% for the 1793 patients discharged with a diagnosis of head/neck injury. Of 671 patients with a discharge diagnosis for the thoracic region, 92.6% returned to work. Of 402 patients with abdominal injury diagnoses, 90.8% returned to work. Of 1603 patients discharged with a diagnosis of extremity injury, the RTW rate was 93.6%. Of 192 patients with a discharge diagnosis of injury in multiple regions, 91.7% returned to work.

**Conclusion:**

Overall, mortality rates were low and RTW rates high in patients who had an ambulance dispatched due to a traffic accident. Those admitted to the ICU had the lowest RTW rate, yet still around 80% returned to work.

## Introduction

Traffic accidents are a leading cause of mortality in the younger population and the World Health Organization (WHO) declared 2011–2020 to be *“The Decade of Action for Road Safety”*, aiming for a reduction of 50% in morbidity and mortality by 2020 [1]. Studies on road traffic injury (RTI) suffers from data unreliability and missing capture [2]. Under-reporting leads to risk of bias, and weakens the potential for generalizability to an entire population. Furthermore, the greater majority of traffic accidents are non-fatal, invoking a need for alternative long-term evaluation. However, only few studies have examined the extent to which survivors suffer from functional impairment and/or how well they recover on a long-term basis, and studies are oftentimes based on specific sub-populations [3–6]. In Denmark, possibilities of such studies are nonpareil due to unified national registries containing a variety of information on all citizens and permanent residents. Specifically, in every ambulance dispatch is recorded in a relevant registry and furthermore, the Danish free health care and EMS system greatly reduces selection bias induced by socioeconomic status.

Return to work (RTW) may be considered as having the capability to continue functioning in society as the individual had prior to an event such as a traffic accident. Accordingly, it has previously been described as a marker of favorable function in studies on trauma, cardiac arrest and heart failure [6–10].

Therefore, the purpose of this study was to examine outcomes after traffic accidents in terms of 1-, 30- and 365-day mortality as well as RTW. We wanted to study outcomes according to International Classification of Disease (ICD-10) discharge diagnoses of anatomical region of injury (ICD-10 Chapter 19 injury classification) including injuries in multiple anatomical regions. Finally, we wanted to study outcome according to whether patients were admitted to the ICU.

Admission to an ICU following a traffic accident may indicate more severe bodily damage [11]. The anatomical site of injury may also impact survival outcomes and function in survivors [12].

## Methods

### Study setting

This study is based on data from the North Denmark Region, an area of approximately 8000km^2^ consisting mainly of suburban (areas with 2500–50,000 citizens) and rural areas (< 2500 citizens), and few urban areas (areas with population > 50,000) and counting 582,413 inhabitants in the last quarter of 2014.

In Denmark, pre- and in-hospital healthcare is financed through taxes, and as such, activation of the EMS and subsequent in-hospital treatment is free of charge. The regional EMS dispatch center has been staffed by healthcare professionals since April 2010, and before April 2010, it was staffed by technical logistics personnel. EMS personnel are primarily trained in basic life support and have two different education levels (an emergency medical technician and an ambulance assistant); this ambulance staff also transports the patients to hospital. In addition, rapid response units staffed by paramedics or mobile emergency care units staffed by an anesthesiologist and a paramedic may also be dispatched to severe emergencies to assist the ambulance staff.

### Study population

We included patients in the North Denmark Region who had an ambulance dispatched following a traffic accident between 4th of April 2006 and 31st December 2014. For patients with more than one ambulance dispatch to different traffic accidents during the study period, we included the latest ambulance dispatch to avoid underestimation of mortality and functional impairment. Patients with a missing or invalid Civil Person Registry number were excluded, as it was not possible to link these patients to other data sources. Furthermore, we excluded patients who were not 18–65 years of age, and patients who were within the specified age-range but not actively working prior to the study.

### Study design, variable and data sources

This study is a registry-based cohort study based on prehospital data from the North Denmark Region from 2006 to 2014. Patients were included using data from the Electronic Prehospital dispatch (amPHI™) database, where each patient transport had a unique dispatch ID. Prehospital data was linked to in-hospital diagnoses, mortality and employment data using the unique Civil Person Registry (social security) number given to each Danish citizen at the time of birth [13]. Patients were considered brought to hospital in cases where a receiving hospital was noted.

In-hospital data, including ICU admittance, comorbidities, length of stay (LoS) and discharge diagnoses was gathered from the National Patient Register. Discharge diagnoses (ICD-10) were assigned if the patient had a hospital admission starting within 30 days following the emergency dispatch, and were grouped according to the anatomical region of injury: (1) head/neck, (2) thorax, (3) abdomen and pelvis, (4) extremities, and (5) multiple regions [14]. Furthermore, patients were stratified according to whether they were admitted to the ICU.

The Charlson comorbidity score was calculated utilizing data from the National Patient Register, and The National Prescription Registry [15, 16].

Socioeconomic status was derived from Statistics Denmark using highest attained educational degree [7, 17]. In accordance with the International Standard Classification of Education (ISCED), patients were divided in three groups: ISCED 0–2 including early childhood education to lower secondary educations; ISCED 3–5 including upper secondary to short-cycle tertiary educations; and ISCED 6–8 including bachelor’s degree to a doctoral degree.

Employment data for analysis of RTW was collected from the Danish National Labor Market Authority [9]. From this database, we were able to identify if patients were on any kind of social benefit on a week-to-week basis, both before the accident and until 1 year following the event. Patients were considered as working (self-supporting) prior to the accident, if they neither on any permanent social benefits in any weeks within the last 5 weeks prior to the accident, nor on temporary social benefits such as sickness leave for more than two of the five-week span. Accordingly, for follow-up, the same five-week span definition was used for one-year rates of RTW.

The study period ended on December 31, 2014, to allow at least 1 year of follow-up for all included patients. The five-week evaluation of employment status was used both at baseline and 1-year follow-up to reduce misclassification, i.e. if sick leave periods during the 5-week span were ≤ 2, patients were classified as working [18]. Patients on State Education Fund grants, maternity leave pay, or leave-of-absence schemes were classified as working. Patients on unemployment benefits, sickness leave pay, early or regular retirement were defined as being on social benefits.

### Availability of data and materials

Data is available from Statistics Denmark research project 4254 and is with access only to approved researchers. https://www.dst.dk/en/TilSalg/Forskningsservice.

Data management was conducted using SAS for Windows, version 9.4 (License required), and analyses using the independent platform R, version 3.5.0 (Open-source).

### Outcome measures

We studied the following outcomes: 1- and 30-day mortality as well as RTW and mortality at one-year follow-up Furthermore, we investigated the influence of sex, age, socioeconomic status and comorbidity prior to the accident on the odds of returning to work.

### Statistical analyses

Categorical data was reported as counts and percentages and continuous data as median with 25–75% percentiles (p25-p75). Descriptive, categorical data was compared with chi-squared test. Cumulative incidences were reported using Aalen-Johansen estimates. Cumulative incidences of RTW are presented considering the competing risk of death. Outcomes were stratified according to admittance to ICU, and location of injury according to the chapter 19 ICD-10 injury classification. Accordingly, pelvic injuries were included in the abdominal category, and spinal injuries were classified by topography. When we examined factors associated with RTW, we treated RTW as a dependent variable in Cox proportional hazard models. Results are presented as cause-specific hazard ratios (HR) with corresponding 95% confidence intervals (CI). We used sex, age, and socioeconomic status (described previously) as exposures, and Charlson comorbidity index was dichotomized to fit the model as 0 versus > 0 because most patients were assumed to be young and healthy as previously shown [19]. Each exposure was adjusted for the other three. Likewise, length of stay was treated binarily as either 0–1 day or more than 1 day to ensure that shorter visits at the hospital crossing midnight were not treated as actual admissions. Age was divided into five groups: 18–29, 30–39, 40–49, 50–59 and 50–65 years of age. Each exposure was analyzed adjusting for the others in a multivariate analysis depicted in Fig. 5. The assumption of proportional hazards was tested and not violated.

## Results

### Patients and characteristics

**Fig. 1:**
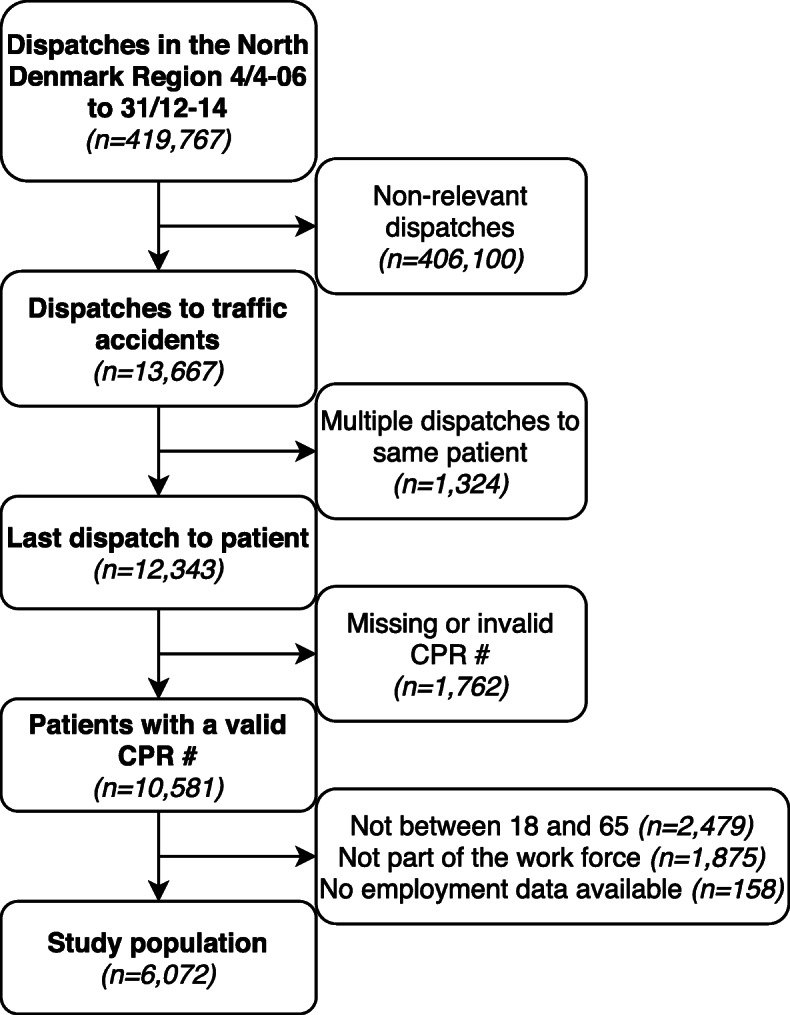
Flowchart of selection of study population. Selection process of patients from prehospital data

We identified 419,767 ambulance dispatches from April 4, 2006 to December 31, 2014, of which 13,667 were marked as dispatches to traffic accidents (Fig. 1).

Of these dispatches, we identified 12,343 individual patients and included the latest ambulance dispatch. A total of 9865 (79.9%) were transported to hospital. In total 6072 patients were between 18 and 65 years of age and employed prior to their accident. Of these patients 3497 (57.6%) were male, and median age was 31.4 (p25-p75; 21.8–44.8). 4,8% (*n* = 290) were admitted to the ICU. Baseline characteristics for all 6072 patients are shown in Table 1.

**Table 1:** Characteristics of study population according to return to work within 1 year

Variable		RTW	No RTW	Dead	Total ***(n*** = 6072)	***P***
**Age**	18–29	2712 (48.2)	76 (21.5)	25 (28.4)	2813 (46.3)	
	30–39	1087 (19.3)	85 (24.0)	11 (12.5)	1183 (19.5)	
	40–49	923 (16.4)	83 (23.4)	19 (21.6)	1025 (16.9)	
	50–59	737 (13.1)	67 (18.9)	22 (25.0)	826 (13.6)	
	60–65	171 (3.0)	43 (12.1)	11 (12.5)	225 (3.7)	< 0.01
Sex*	Male	3219 (57.2)	209 (59.0)	69 (78.4)	3497 (57.6)	< 0.01
**Socioeconomic status**^**a**^	1	2413 (42.9)	179	42	2634 (43.4)	
	2	2229 (39.6)	129	35	2393 (39.4)	
	3	733 (13.0)	28	10	771 (12.7)	0.018
	*missing*	255 (4.5)	≤20	≤3		
**Charlson score**	0	5534 (98.3)	326 (92.1)	82 (93.2)	5942 (97.9)	
	> 0	96 (1.7)	28 (7.9)	6 (6.8)	130 (2.1)	< 0.01
**Dispatch priority**	1	5442 (96.7)	332 (93.8)	88 (100.0)	5862 (96.5)	
**to accident**^**b**^	2	188 (3.3)	22 (6.2)	0 (0.0)	210 (3.5)	< 0.01
**Return to**	0	186 (3.4)	6 (1.7)	8 (9.3)	200 (3.3)	
**hospital priorty**^**b**^	1	1289 (23.3)	125	36	1450 (24.3)	
	2	4064 (73.4)	216	42	4322 (72.4)	< 0.01
	*missing*	91	< 10	< 10		
**Admitted to ICU¤**	Yes	2469 (4.8)	39 (11)	9 (10.2)	317 (5.2)	< 0.01
**LoS¤ > 1 day**	Yes	658 (11.7)	100 (28.2)	9 (10.2)	767 (12.6)	< 0.01
**1- day mortality**	Yes	≤3	≤3	59 (67.0)	59 (0.97)	< 0.01
**30- day mortality**	Yes	≤3	≤3	73 (83.0)	76 (1.3)	< 0.01

*¤Abbreviations:* ICU = Intensive care unit, LoS = Length of Stay; *count and column percentage (%).^a^International Standard Classification of Education (ISCED), group 1 = ISCED 0–2, group 2 = ISCED 3–5, group 3 = ISCED 6–8.^b^Dispatch priority is grouped by level of emergency, 1 being an emergent dispatch and 2 being only sub-emergent. Priority 0 is “no-return journey” and most likely account for cases where patients were able to transport themselves to the receiving hospital. In some columns percentages was left out, and small numbers masked to avoid risk of identifying individuals

### One-, 30- and 365-day mortality

Of 6072 patients in our study population, 59 (1%) died within 1 day and 76 (1.3%) within 30 days. 88 (1.5%) died within a year.

30-day mortality was 1.7% for the 290 patients admitted to the ICU, and 1.2% for the remaining 5782 patients.

### Return to work

**Fig. 2:**
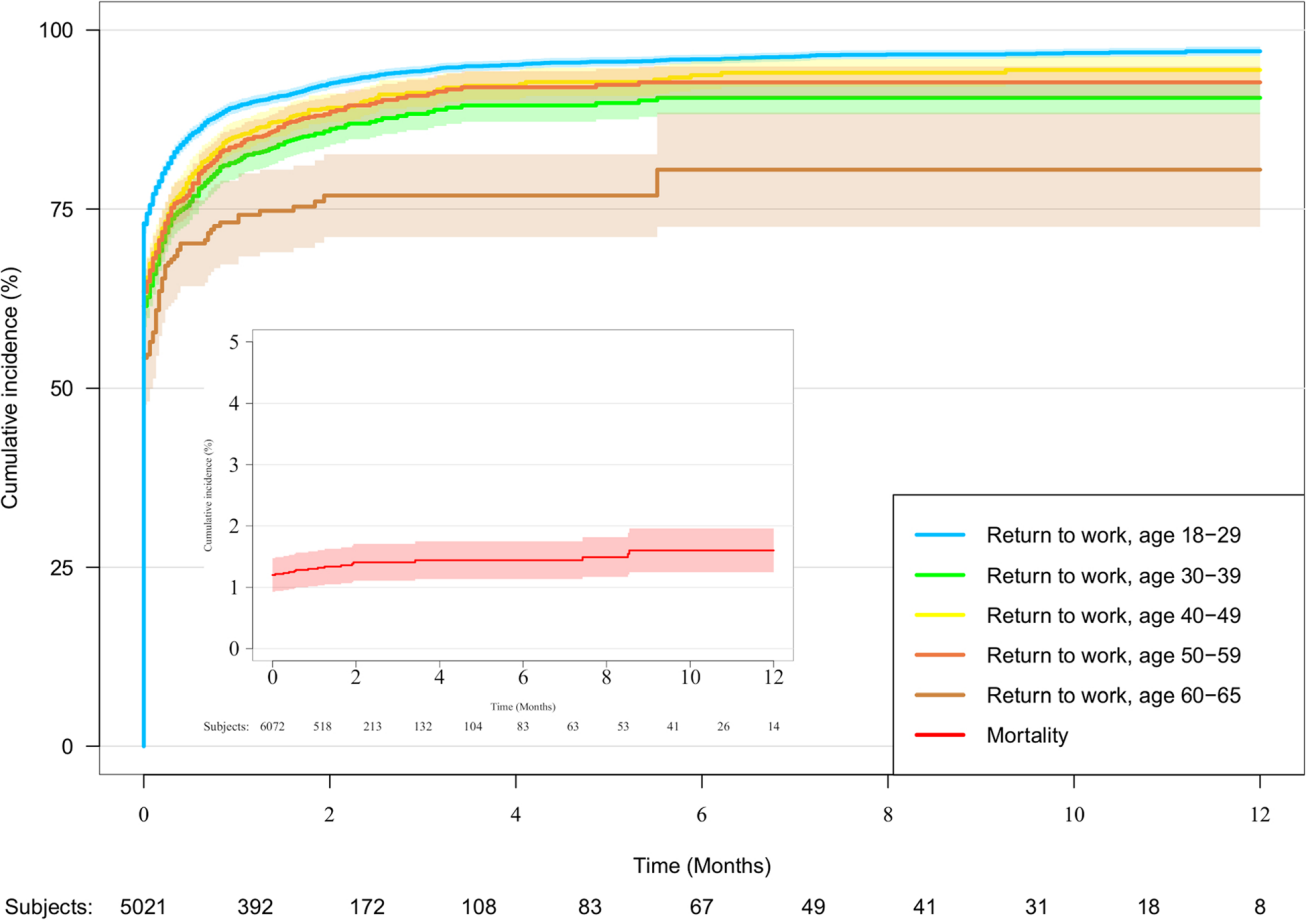
Cumulative incidences of RTW and mortality in study population. Cumulative incidences of RTW and mortality in study population (*n* = 6072). The blue lines show the cumulative incidence of RTW within one year after traffic accident. The red line depicts mortality. The shaded areas depict the 95% confidence intervals. Vertical axis: Cumulative incidence. Horizontal axis: Time (months)

Within the study population, RTW rate was 96.4% for the age group 18–29, 91.9% for the 30–39 year-olds, 90.1% for the 40–49 year-olds, 89.2% for the 50–59 year-olds and 76% for the 60–65 age group, as shown in Fig. 2.

**Fig. 3:**
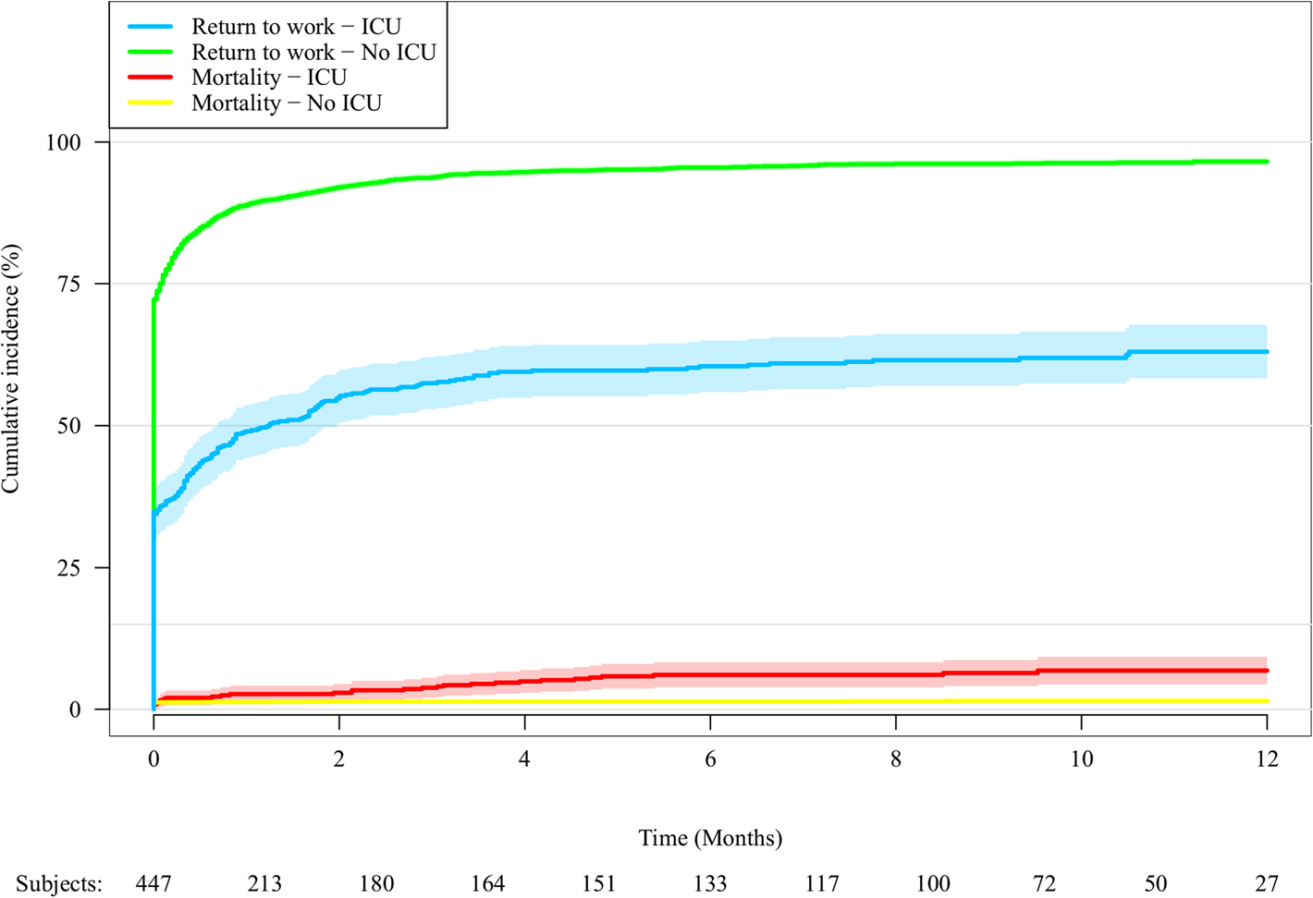
Cumulative incidences of RTW and mortality in ICU admitted patients. Cumulative incidences of RTW and mortality in ICU admitted patients (*n* = 290) versus non-ICU admitted patients (*n* = 5782). The blue and green lines depict the cumulative incidences of RTW within one year after traffic accident. The red and yellow lines depict mortality. The shaded areas depict the 95% confidence intervals. Vertical axis: Cumulative incidence. Horizontal axis: Time (months)

RTW was 84.7% among 317 ICU-admitted patients versus 93.2% for the remaining 5755 patients, as illustrated in Fig. 3.

**Fig. 4:**
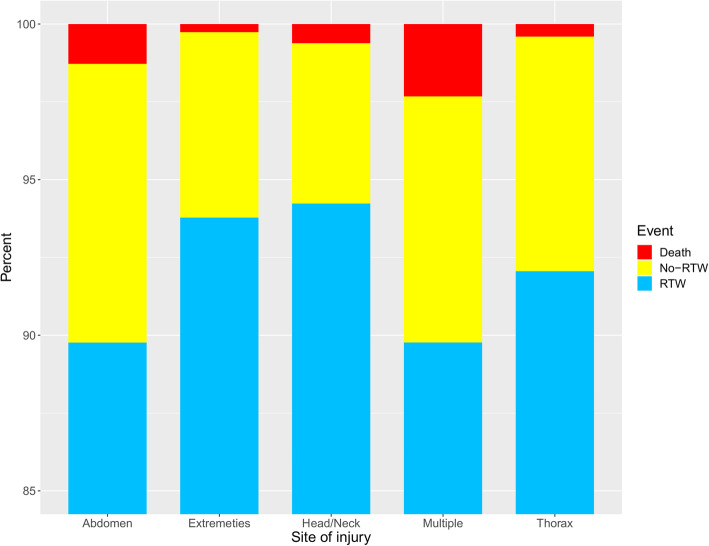
Cumulative incidence of return to work, no-return to work and death according to area of discharge diagnosis within a year. Percentage RTW, no- RTW and death according to area of discharge diagnosis respectively head/neck (*n* = 2272), thorax (*n* = 743), abdomen (*n* = 469), extremities (*n* = 2686) and multiple regions (*n* = 215). Blue indicates return to work, yellow indicates no-return to work, and red indicates death. Vertical axis: Percentage. Horizontal axis: Site of injury

RTW rate was 94.3% for 1807 patients discharged with a diagnosis of head/neck injury. Of 689 patients with a discharge diagnosis referring to the thoracic region, 92.6% returned to work. Of 408 patients with abdominal injury diagnoses, 90.2% returned to work. Of 1621 patients discharged with a diagnosis of extremity injury, the RTW rate was 93.8%. Of 204 patients with discharge diagnosis of injury in multiple regions, 90.7% returned to work. These results are illustrated in Fig. 4.

**Fig. 5:**
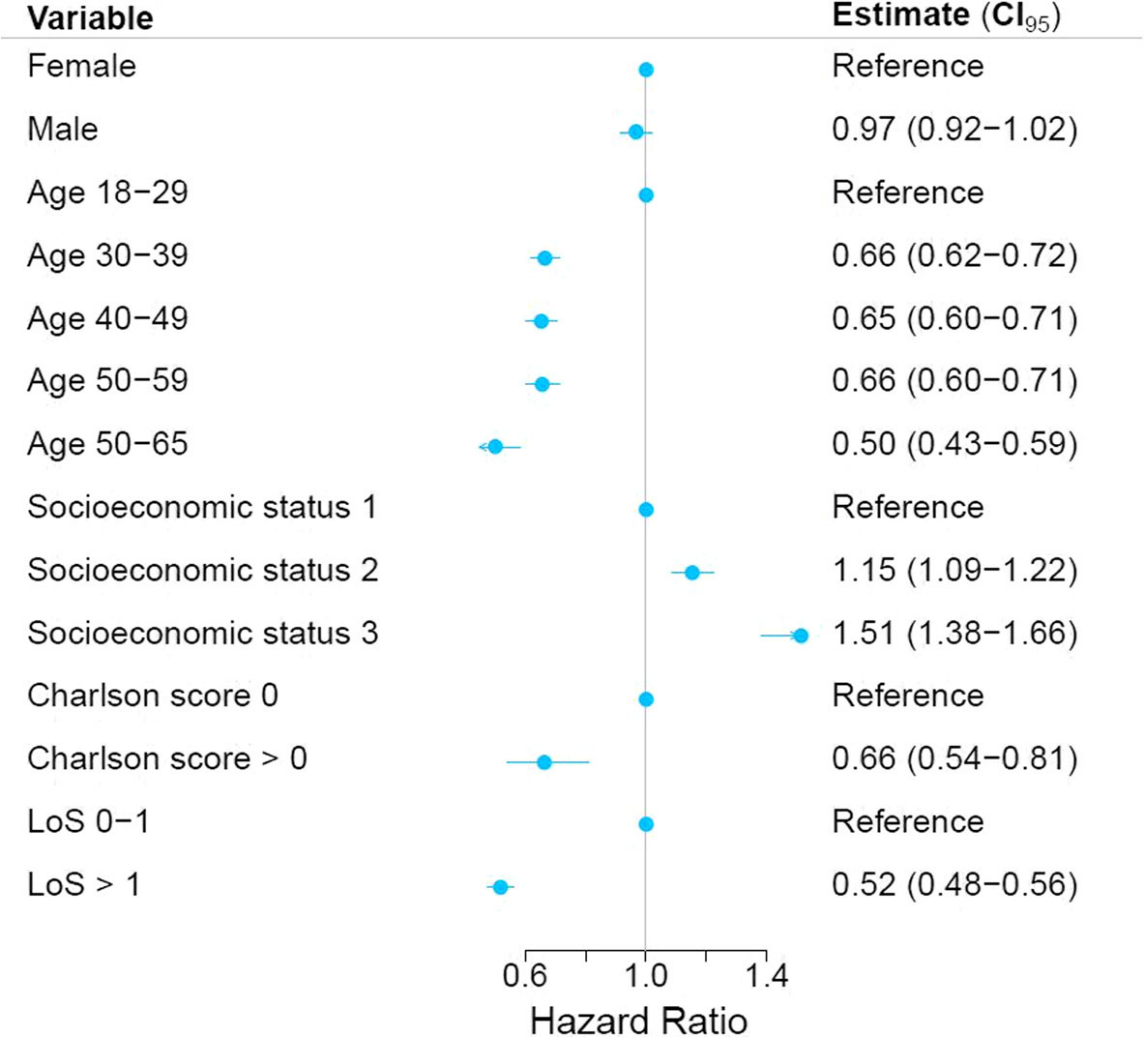
Association between sex, age, socioeconomic status and Charlson comorbidity score and return to work. Blue dot indicates hazard ratio of RTW, and line indicates 95% confidence intervals. Socioeconomic status refers to International Standard Classification of Education (ISCED), group 1 = ISCED 0–2, group 2 = ISCED 3–5, group 3 = ISCED 6–8

Older age group, having a Charlson score > 0 and a hospital admission longer than 1 day was significantly associated with lower hazard rate of returning to work. Compared to ages 18–29 the HR for age group 30–39 was 0.66 (95% CI 0.62–0.72), for age group 40–49 it was 0.65 (CI 0.60–0.71), 50–59 years 0.66 (CI 0.60–0.71) and 50–65 years 0.50 (CI 0.43–0.59). The HR for a Charlson score > 0 was 0.66 (CI 0.54–0.81) and for LoS > 1 day it was 0.52 (CI 0.48–0.56). The hazard ratio for returning to work for individuals with socioeconomic status level 2 was a HR of RTW of 1.15 (95% CI 1.09–1.22), whilst individuals with socioeconomic status level 3 had a HR of 1.51 (95% CI 1.38–1.66) when compared to socioeconomic status level 1. Thus, having a better socioeconomic status prior to accident increased odds of returning to work afterwards. This is depicted in Fig. 5.

## Discussion

In this study of traffic accidents in the North Denmark Region from 2006 to 2014, the overall 30-day mortality was low at 1.3% and the overall RTW rate high at 92.7%. RTW rates were lowest amongst more severely injured patients who were admitted to the ICU. However, even amongst these more severely injured patients, RTW rates were a mere 8% lower at around 85%.

Traffic accidents constitute a leading cause of death in the younger population. Accordingly, WHO has declared this current decade to be the “Decade of Action for Road Safety”, with campaigns aiming at reducing deaths and injuries following traffic accidents [1, 20–22]. Most of the contemporary literature on traffic accidents is from low-income countries where mortality due to several factors is significantly higher and not comparable to this study [1, 2, 23]. In higher-income countries, 30-day mortality rates range from 1 to 5%, in line with our study findings:

Recent national, Danish numbers show a mortality of 3.0 pr. 100.000 inhabitants in 2018, which corresponds to statistics for similar countries (Sweden 3.2, Germany 4, United Kingdom 2,8). This speaks to the external validity of the study, at least within Northern Europe [24]. Although mortality rates are mainly low, the extent to which discharged survivors have functional impairments is scarcely studied [5]. RTW has previously been evaluated as a proxy for favorable functional outcome in out-of-hospital cardiac arrest (OHCA) survivors and in heart failure patients [7, 8]. Likewise the employment registry has previously been validated, with a positive predictive value of 98.2% for actual employment for patients who were registered as working (self-supporting) [25]. In this study, the majority of patients involved in traffic accidents are of working age, making RTW a suitable marker for good functional outcome.

Most patients had the capability to return to work within 1 year after their traffic accident. This finding is in line with a RTW rate of 73.1% in patients discharged from hospital after trauma-related injuries [2, 9, 26]. Present study also included non-survivors, and has the advantage of being conductedona population scale, where even patients with a low socioeconomic status and poor connection to the work force, due to the free health care system, are included. These patients included those admitted to the ICU and patients with higher Charlson comorbidity score and those with lower socioeconomic status.

Conversely, it was anticipated that many patients would be able to RTW, given a wide spectrum of injury from minor injury, temporary sickness leave (i.e. following concussion) to presumably only few patients with multiple injuries requiring ICU admittance.

Factors associated with returning to work were lower age, lower Charlson comorbidity index and higher socioeconomic status. This is in line with previous findings of factors associated with RTW in more selected populations, of OHCA, stroke and acute respiratory distress syndrome respectively [7, 27, 28]. In these studies, association between sex and RTW varies. Correspondingly in this study, female sex showed only a slight association. Underlying causes for these associations can only be speculated upon with the present data. It is plausible, that higher socioeconomic status in general increases the odds of having a less physically demanding occupation, and therefore easier re-employment. Furthermore, higher socioeconomic status might increase the chance of the individual’s social network helping either directly or indirectly with returning to work. Furthermore, higher age and more comorbidity might cause employment prior to RTI to be less stable, requiring less negative impact to result in unemployment.

Some patients had missing data for hospital return, or had no recorded return journey to hospital. This may either indicate: on one hand that complaints and/or injuries were of minor magnitude or even non-existing and not requiring any hospital contact or the other extreme being that patients already were dead at the time of EMS arrival. Nonetheless, the vast majority survived and were able to return to work, indicating that outcomes following traffic accidents generally are good in the North Denmark Region, and by extension also likely in other regions in Denmark and in other developed countries.

### Limitations

This study has some limitations. First, it is observational in nature, meaning that no causal relationships can be made. Second, the basis for inclusion in this study is dependent on registration of a traffic accident in the prehospital dispatch database, which was mainly filled in by logistic staff. Third, missing or invalid Civil Personal registration (social security) numbers on several patients prevented us from analyzing outcomes for these patients. Fourth, using a cut-off at 24-h might underestimate 1-day mortality, as death is not always registered promptly after it has occurred. Fifth, capture of un-employment may not be complete, as a person can choose not to receive social benefit whilst unemployed. It is expected that this number is very low [25]. Sixth, we did not have scoring methods such as an Abbreviated Injury Score and a final Injury Severity Score available, which hampers comparison with trauma-specific studies. In continuation, few patients were severely injured as measured by admittance to the ICU. Further evaluation of outcomes including RTW in relation to such procedures, as well as in relation to an in-hospital injury severity score, comorbidities and other potential predictors of outcome among more severely injured patients is warranted.

## Conclusion

Overall, mortality rates were low and RTW rates high, and whilst RTW rates were lower amongst more severely injured patients who were admitted to the ICU, RTW rates were still very high. While further studies of functional outcome in patients involved in road traffic accidents are warranted, this study indicates that even though globally RTI is a significant problem, patients involved in traffic accidents in the North Denmark Region seem to have low mortality and high RTW rates.

## Data Availability

The data that support the findings of this study are available from Electronic Prehospital dispatch (amPHI™) database in the North Denmark Region, the Danish Civil Person Registry, the Danish National Patient registry and the Danish National Prescription registry respectively, but restrictions apply to the availability of these data, which were used under license for the current study, and so are not publicly available. Data are however available from the authors upon reasonable request and with permission from the given registry. Data access and analysis was approved by the Danish Data Protection Agency (reference 2007-58-0015, GEH-2014-019, I-suite 02737).

## Abbreviations

EMS: Emergency medical service
ICU: Intensive care unit
ISCED: International Standard Classification of Education
OHCA: Out-of-hospital cardiac arrest
RTI: Road traffic injury
RTW: Return to work
WHO: World Health Organization
